# Evaluation of EUCAST rapid antimicrobial susceptibility testing (RAST) for positive blood cultures in clinical practice using a total lab automation

**DOI:** 10.1007/s10096-020-03846-3

**Published:** 2020-02-28

**Authors:** Jasmin Kaur Jasuja, Stefan Zimmermann, Irene Burckhardt

**Affiliations:** Department for Infectious Diseases, University Hospital Heidelberg, Heidelberg, Germany

**Keywords:** RAST, Time to report, Total lab automation (TLA), Blood culture, EUCAST

## Abstract

Our objective was to evaluate EUCAST’s ‘rapid antimicrobial susceptibility testing’ (RAST) directly from positive blood culture that delivers antimicrobial results within 6 h for *Staphylococcus aureus*, *Enterococcus* spp., *Escherichia coli*, *Klebsiella pneumoniae* and *Pseudomonas aeruginosa*, using total lab automation. Zone diameters from RAST were compared with MIC results. Furthermore, its influence on time to report was investigated. RAST was performed to all positive aerobic and anaerobic blood culture bottles by subculturing them, i.e. onto Mueller-Hinton agar and adding six antibiotic discs covering Gram-negative and Gram-positive therapy (cefoxitin, ampicillin, vancomycin, piperacillin/tazobactam, meropenem and ciprofloxacin). RAST was automatically imaged after 6 h. Zone sizes were measured using a TLA software tool and interpreted according to EUCAST clinical breakpoints. Bacteria were identified using MALDI-TOF MS and MIC results were determined using Vitek2 panels. Categorial agreement between agar diffusion and MIC results was investigated. Additionally, time to RAST and time to Vitek were compared for 100 isolates (20 per species). Between November 2018 and April 2019, 3313 positive mono-bacterial blood culture bottles were collected of which 894 bottles with RAST-validated species were investigated. Among these bottles, 2029 individual antibiotic measurements were compared with MIC results from Vitek2 and 14 very major, 28 major and 12 minor errors were found. A median reduction of 17:30 h in time to report was observed. Introduction of RAST with automatic TLA imaging function could reduce time to report by 17:30 h. Excellent accordance between zone diameter and MIC results, particularly for cefoxitin, vancomycin and meropenem, was observed, but drawbacks due to ATU were seen.

**Electronic supplementary material:**

The online version of this article (10.1007/s10096-020-03846-3) contains supplementary material, which is available to authorized users.

## Introduction

The rapid diagnosis of sepsis plays a major role since bloodstream infections are considered as an important cause of morbidity and mortality in hospitalised patients [1]. Administering inappropriate antibiotic therapy, especially to severe ill and septic patients, has been negatively associated with the patient’s outcome [2]. Furthermore, each hour of delay in the administration of correct antibiosis is associated with a decrease in survival of septic patients [2]. To improve correct empirical antimicrobial treatment, rapid reporting of microscopy results has shown the greatest impact on appropriate therapy [3]. This is why improved processing of positive blood cultures, its rapid identification and AST, is essential for clinicians in order to treat patients with bloodstream infection properly [4]. Furthermore, it is assumed that the addition of antibiotic stewardship programs (ASP) to rapid techniques may bring out greater benefits than ASP and microbial diagnostics alone, respectively, and rapid tests could also contribute to the early interruption of unnecessary treatment [1, 5].

Traditionally, the laboratory process is divided into pre-analytic, analytic and post-analytic phases. The number of inoculated blood culture bottles per patient, blood volume per bottle, bacterial load and delay in sample transfer pertains to pre-analytic aspects that may delay the start of incubation and correspondingly the time to positivity. For analytical workflow, the incubation time and performing Gram stain, ID and AST play a major role. Microbial growth in blood culture bottles usually takes 24–48 h. Identification with MALDI-TOF MS can be achieved as soon as 3–4 h after start of subculture [6–8]. Numerous studies have shown the performance of MALDI-TOF MS directly from positive blood culture broth with results available within few hours, but have been hampered by a poor accuracy for Gram-positive bacteria [9]. The same applies to AST directly performed from positive blood culture bottles with Vitek2 and Phoenix [9–11]. However, current guideline-defined AST protocols require one overnight incubation step. Such overnight procedures may end fatally for septic patients, particularly those infected with multi-resistant pathogens [5]. Therefore, molecular assays like RT-PCR have been introduced for the rapid identification of bacteria and the simultaneous detection of specific resistance genes directly from positive blood culture broth [12]. Still, assays are limited to available primers and probes and have not been sufficiently tested for routine use [12].

Our approach aimed to reduce the time to report AST of positive blood cultures. Therefore, the new method ‘rapid antimicrobial susceptibility testing’ (RAST) directly feasible from positive blood culture established by EUCAST was introduced on total lab automation (TLA, BD Kiestra™) for common and validated pathogens in bloodstream infections, namely *Staphylococcus aureus*, *Enterococcus* spp., *Escherichia coli*, *Klebsiella pneumoniae* and *Pseudomonas aeruginosa*. We assessed the time saved by reporting RAST as preliminary AST results in order to evaluate RAST in clinical practice. Zone diameters were also compared with MIC results obtained from Vitek2 to determine ‘very major’, ‘major’ and ‘minor’ errors and to observe to which extent MIC results could be predicted.

## Material and methods

### Setting

The study was performed at the Department for Infectious Diseases at the University Hospital Heidelberg, Germany. We offer a 12-h service period on weekdays and a 10-h service period on weekends and public holidays.

Our analysis included aerobic and anaerobic blood culture bottles (BD BACTEC™ Plus Aerobic/F, BD BACTEC™ Plus Anaerobic/F) that signalled positivity between 1 November 2018 and 30 April 2019. PED blood culture bottles and those inoculated with other materials than blood (e.g. joint fluid) were excluded. The pair of an aerobic and anaerobic blood culture bottle was given the same case number, but for analysis, bottles were treated individually. After arrival at our laboratory, blood culture bottles were incubated in the BD BACTEC™ FX instrument for up to 7 days or until they signalled positive.

### Processing of positive blood culture bottles

**Fig. 1:**
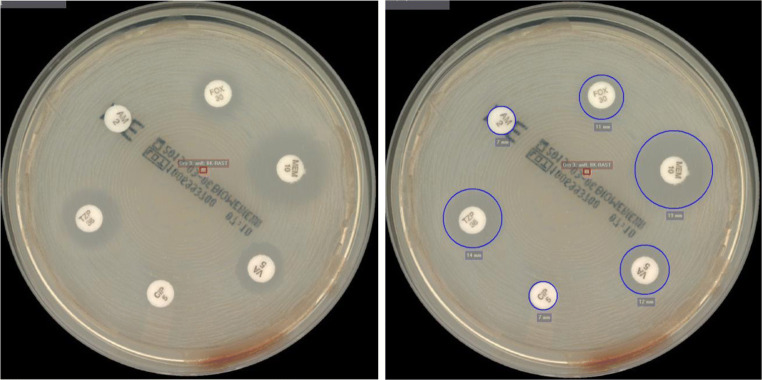
Images of RAST plate of methicillin-resistant *Staphylococcus aureus* (MRSA) without (left) and with (right) measured inhibition zones automatically taken with the photo lab function of the total lab automation (TLA, BD Kiestra™) at the Department for Infectious Diseases at the University Hospital Heidelberg, Germany. The inhibition zone for cefoxitin (FOX) is measured with 11 mm. According to the EUCAST zone diameter breakpoints for RAST directly from blood culture bottles, this zone diameter is within resistant category and, hence, indicates an MRSA

Bottles which signalled positive during operational time were processed using the semi-automatic part of a TLA system: a Gram slide was prepared, subcultures were done on blood agar (Columbia agar, 5% sheep blood, BD), chocolate agar (BioMérieux), MacConkey agar (BioMérieux) and in case of an anaerobic bottle additionally on Schaedler/KV agar (5% sheep blood, BD). Streaking was done using the rolling bead technology of the InoqulA module. Furthermore, a rapid antimicrobial susceptibility test (RAST) plate was prepared following the EUCAST instructions [13]. Shortly, 150 μL of blood culture bottle fluid was applied to Mueller-Hinton E agar (BioMérieux) and streaked using streaking pattern no 1, available in the InoqulA software. Six discs were applied to the MHE plate: cefoxitin (30 μg, BD), ampicillin (2 μg, BD), vancomycin (5 μg, BD), piperacillin/tazobactam (30/6 μg, BD), meropenem (10 μg, BD) and ciprofloxacin (5 μg, BD). All aerobic plates were sent to the incubators (35 °C, O_2_: RAST plate, 5% CO_2_: blood agar, chocolate agar, MacConkey agar) of the TLA (ReadA Compact) and imaged after 6 h (all plates) and 23 h (all plates except RAST plate) (Fig. 1). Anaerobic plates were incubated in an anaerobic jar in an external incubator and read together with the aerobic plates.

Identification was done using MALDI-TOF using 6-h growth. Inhibition zones were manually measured by positioning zone circles using a software of the TLA and interpreted according to the EUCAST guidelines for RAST [14]. Additionally, MICs were determined using suitable Vitek2 panels. Gram slides and RAST were performed for all positive blood culture bottles. MIC determination was done once per patient and bacterial species. Wards were notified of the positive blood culture and the Gram slide results by telephone and electronic reporting. A preliminary report with ID and RAST and final report were sent separately. In case of MIC determination, an additional report was generated. In case of blood culture bottle signalling positivity in the afternoon, images of subcultures and RAST were taken outside operational time and were interpreted in the next morning after performing MALDI-TOF MS. RAST was electronically reported afterwards and MIC results were prepared for the next day. Successful quality control was done with *E. coli* and *S. aureus*.

### Comparison of RAST results and Vitek2 results

For data evaluation, we used the terms categorial agreement, very major, major and minor errors, as defined by Cumitech [12]. Very major errors (VME) were defined as susceptible response by the new AST (here: RAST) while the reference method (here: Vitek2) resulted in resistant response. Major errors (ME) were defined as resistant response in the new AST while the reference method indicated a susceptible response. Minor errors (MinE) were observed when either the new AST or the reference method indicated an intermediate response and the other one indicated a susceptible or resistant response. Instead of an intermediate category, EUCAST introduced the concept of ‘area of technical uncertainty’ (ATU) ‘where the separation between susceptibility categories (S, I, R) is poor’ [14]. ATU results were not interpreted and minor errors could only arise when comparing susceptible and resistant RAST to intermediate Vitek results.

According to Cumitech, categorial agreement should be ≥ 90%, VME and ME rate should be ≤ 3%, respectively. The combined performance rate for ME and MinE rate is supposed to be ≤ 7% [15].

To ease the analysis and since we wanted to predict MICs with RAST, we assumed that Vitek2 results were 100% correct.

VME and ME rates were calculated based on the number of total resistant and total susceptible isolates, respectively. The categorial agreement and the MinE rate were calculated based on the number of all tested isolates without the isolates with an ATU result (RAST). VME, ME and MinE rates were calculated for each drug and each drug-species combination.

Data from previous years implied that a minimum amount of 200 *S. aureus*, 200 *Enterococcus* spp., 300 *E. coli*, 100 *K. pneumoniae* and 30 *P. aeruginosa* seemed reasonable to achieve during the intended study period of 6 months. Analysis was done batch-wise and was stopped as soon as the aimed amount was reached or exceeded.

For an overview of evaluated antibiotics and species see Table 1.

**Table 1:** Overview of all sent blood culture bottles, positive blood culture bottles and RAST-validated pathogens within the time period of 1 November 2018 to 30 April 2019 during introduction of RAST on total lab automation (TLA, BD Kiestra™) at the Department for Infectious Diseases at the University Hospital Heidelberg, Germany. Overall, 33,246 aerobic and anaerobic blood culture bottles were sent, of which 3313 bottles became positive and were mono-bacterial. A total of 1482 positive blood culture bottles were identified with RAST-validated species of which 894 bottles were analysed and compared with MIC results. In total, 2029 individual antibiotic measurements were analysed. Time to RAST (TTR) and time to Vitek (TTV) were analysed for 100 isolates, 20 isolates per species. A total of 1979 positive blood culture bottles were found with other species. Species with ≤ 20 isolates were categorised

Time period	1 November 2018–30 April 2019
Overall blood cultures - Aerobic bottles - Anaerobic bottles	*n* = 33,246 *n* = 16,628 n = 16,618
Positive blood culture bottles - Aerobic bottles - Anaerobic bottles - False-positive bottles - Blood culture bottles with multiple pathogens	*n* = 3461 *n* = 1748 *n* = 1713 *n* = 78 *n* = 73
- Positive blood culture bottles with mono-bacterial growth	*n* = 3313
Pathogens for which RAST was applicable	*n* = 1482
- Thereof analysed species (for RAST-Vitek comparison and evaluation of VME, ME and MinE)	*n* = 894
Thereof analysed individual antibiotic measurements	*n* = 2029
*S. aureus* - MSSA - MRSA	*n* = 221 *n* = 212 *n* = 9
*Enterococcus* spp. - *E. faecalis* - *E. faecium* - *VRE*	*n* = 211 *n* = 72 *n* = 71 *n* = 68
*E. coli*	*n* = 319
*K. pneumoniae*	*n* = 113
*P. aeruginosa*	*n* = 30
Comparison ‘time to RAST’ and ‘time to Vitek’	*n* = 100 (20 isolates per pathogen)
Other species than those validated for RAST	*n* = 1979
*S. epidermidis*	*n* = 634
*Citrobacter* spp.	*n* = 19
*Enterobacter* spp.	*n* = 59
*Serratia marcescens*	*n* = 40
*Klebsiella* spp. (other than *K. pneumoniae*)	*n* = 39
*Proteus mirabilis*	*n* = 36
*Candida* spp.	*n* = 62
*Clostridium* spp.	*n* = 19
Coagulase-negative *Staphylococcus* (*S. capitis*, *S. caprae*, *S. haemolyticus*, *S. hominis*, *S. lugdunensis*, *S. petrasii*, *S. pettenkoferi*, *S. saccharolyticus*, *S. warneri*)	*n* = 174
Gram-positive bacilli (*Actinomyces neuii*, *Bacillus cereus*, *Blautia coccoides*, *Brevibacterium celere*, *Corynebacterium afermentans*, *Corynebacterium jeikeium*, *Corynebacterium kroppenstedtii*, *Corynebacterium* spp., *Cutibacterium acnes*, *Dermabacter hominis*, *Lactobacillus* spp.)	*n* = 38
*Bacteroides* spp. (*B. caccae*, *B. fragilis*, *B. ovatus*, *B. vulgatus*)	*n* = 19
*Streptococcus pneumoniae*	*n* = 43
*Streptococcus* spp. (*S. agalactiae*, *S. anginosus*, *S. canis*, *S. constellatus*, *S. dysgalactiae*, *S. gallolyticus*, *S. gordonii*, *S. intermedius*, *S. lutetiensis*, *S. mitis*, *S. oralis*, *S. parasanguinis*, *S. pyogenes*, *S. salivarius*, *S. sanguinis*)	*n* = 182
Other Gram-positive cocci(*Aerococcus urinae*, *Enterococcus casseliflavus*, *Parvimonas* spp.)	*n* = 13
Other *Enterobacterales* (*Hafnia alvei*, *Leclercia adecarboxylata*, *Morganella morganii*, *Pantoea septica*, *Providencia stuartii*, *Salmonella typhi*)	*n* = 17
Other Gram-negative pathogens (*Acinetobacter Iwoffii*, *Aeromonas caviae*, *Aggregatibacter actinomycetemcomitans*, *Campylobacter jejuni*, *Haemophilus influenzae*, *Neisseria perflava*, *Stenotrophomonas maltophilia*, *Sutterella wadsworthensis*)	*n* = 24

### Performance data analysis

**Fig. 2:**
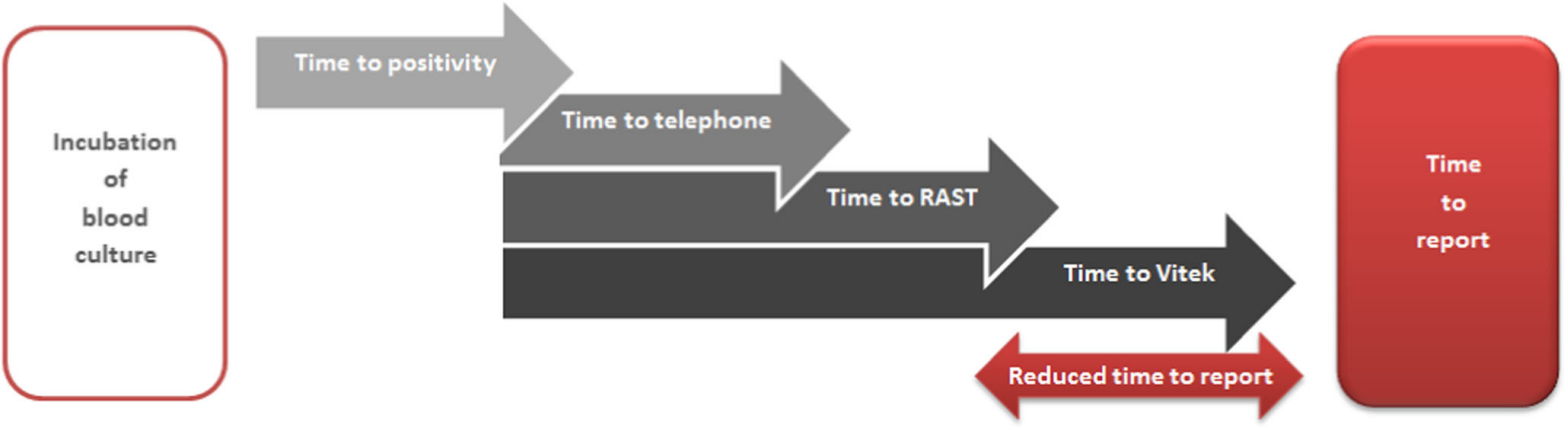
Timeline of blood culture procedure after introducing RAST on total lab automation (TLA, BD Kiestra™) at the Department for Infectious Diseases at the University Hospital Heidelberg, Germany. As soon as a blood culture bottle signalled positive (= time to positivity (TTP)), Gram stain slide, RAST plate and subcultures were prepared. The microscopy results were reported to the attending physician (= time to telephone (TTT)) and a written report was sent electronically. After 6 h, RAST and subcultured plates were automatically imaged and bacteria were identified via MALDI-TOF MS. Written results of RAST and ID were reported (= time to RAST (TTR)). On the upcoming day Vitek2 results were reported (= time to Vitek (TTV)). The interval between TTP and TTV was defined as ‘reduced time to report’

The following data were extracted from the LIS and the following intervals were calculated (Fig. 2):

- Time to positivity (TTP): timepoint of start of incubation ➔ timepoint of positivity
- Time to RAST (TTR): timepoint of positivity ➔ timepoint of RAST report
- Time to Vitek (TTV): timepoint of positivity ➔ timepoint of final Vitek report

Since timepoint of RAST report and timepoint of Vitek2 report had to be retrieved manually to gain information on the time interval of ‘reduced time to report’, the amount of analysed datasets was restricted to 100 bottles. The timepoint of final Vitek report was also the timepoint of reporting final results to the wards electronically.

### Statistical analysis

Data on timepoints and susceptibility test results were obtained from our LIS (SwissLab) and analysed with Microsoft Excel 2010.

## Results

### Overview study specimens

During the 6-month study period from 1 November 2018 to 30 April 2019, a total of 33,246 blood culture bottles were registered (Table 1). Thereof, 3461 (10.4%) bottles signalled positive of which 73 bottles (2.1%) showed poly-microbial growth after 6 h and 78 bottles (2.3%) were false positive, so that 3313 positive mono-bacterial blood culture bottles could be evaluated. 74.0%, 91.6% and 96.7% of bottles became positive within 24 h, 48 h and 72 h after start of incubation. 44.8% of bottles signalled positive during working hours; 55.2% of bottles signalled positive outside the service period.

### Accordance RAST-Vitek

For 894 bottles, the categorical interpretations (susceptible/resistant) of RAST were compared with the respective Vitek2 results. That included 221 isolates of MSSA/MRSA, 211 isolates of *Enterococcus* spp., 319 isolates of *E. coli*, 113 isolates of *K. pneumoniae* and 30 isolates of *P. aeruginosa* (Table 2) and resulted in 2029 individual measurements (221× cefoxitin for *S. aureus*, 211× ampicillin and 211× vancomycin for *Enterococcus* spp., 462× piperacillin/tazobactam, ciprofloxacin and meropenem for *E. coli*, *K. pneumoniae* and *P. aeruginosa*, respectively).

**Table 2:** Interpretation of EUCAST developed rapid antimicrobial susceptibility testing (RAST) directly feasible from positive blood culture for a total of 894 positive blood culture bottles at the Department for Infectious Diseases at the University Hospital Heidelberg, Germany. Inhibition zones from RAST were compared with MIC results obtained from Vitek2. Very major errors (VME), major errors (ME) and minor errors (MinE) were calculated. The EUCAST inhibition zone interpretation guidelines for RAST do not contain an intermediate response. Instead, the concept of ‘area of technical uncertainty’ (ATU) was introduced. Results which fall into the ATU category cannot be used to predict susceptibility or resistance. For determining VME and ME, isolates with ATU interpretation were excluded. MinE were only determined for susceptible and resistant RAST response to intermediate Vitek results. (*S*, susceptible; *R*, resistant; *ATU*, area of technical uncertainty)

		RAST interpretation			Vitek MIC results			Errors		
		*S*	ATU	*R*	*S*	*I*	*R*	VME	ME	MinE
*S. aureus* (*n*=221)	Cefoxitin	95.0% (210/221)	≤ 1% (1/221)	4.5% (10/221)	95.9% (212/221)	/	4.1% (9/221)	/	≤ 1% (1/212)	/
*Enterococcus spp.* (*n*=211)	Ampicillin	34.6% (73/211)	/	65.4% (138/211)	34.1% (72/211)	/	65.9% (139/211)	≤ 1% (1/139)	/	/
	Vancomycin	64.9% (137/211)	/	35.1% (74/211)	67.8% (143/211)	/	32.2% (68/211)	/	4.2% (6/143)	/
*E. coli* (*n*=319)	Piperacillin/Tazobactam	49.2% (157/319)	41.4% (132/319)	9.4% (30/319)	92.8% (296/319)	/	7.2% (23/319)	/	2.7% (8/296)	/
	Ciprofloxacin	66.1% (211/319)	6.6% (21/319)	27.3% (87/319)	73.7% (235/319)	1.3% (4/319)	25.0% (80/319)	/	2.6% (6/235)	≤ 1% (3/298)
	Meropenem	100% (319/319)	/	/	100% (319/319)	/	/	/	/	/
*K. pneumoniae* (*n*=113)	Piperacillin/ Tazobactam	40.7% (46/113)	37.2% (42/113)	22.1% (25/113)	68.1% (77/113)	8.9% (10/113)	23.0% (26/113)	7.7% (2/26)	5.2% (4/77)	7.0.% (5/71)
	Ciprofloxacin	69.9% (79/113)	17.7% (20/113)	12.4% (14/113)	82.3% (93/113)	≤ 1% (1/113)	16.8% (19/113)	21.1% (4/19)	2.2% (2/93)	/
	Meropenem	100% (113/113)	/	/	100% (113/113)	/	/	/	/	/
*P. aeruginosa* (*n*=30)	Piperacillin/ Tazobactam	36.7% (11/30)	43.3% (13/30)	20.0% (6/30)	56.7% (17/30)	/	43.3% (13/30)	5.9% (1/13)	5.9% (1/17)	/
	Ciprofloxacin	66.7% (20/30)	23.3% (7/30)	10.0% (3/30)	50.0% (15/30)	/	50.0% (15/30)	40.0% (6/15)	/	/
	Meropenem	43.3% (13/30)	30.0% (9/30)	26.7% (8/30)	43.3% (13/30)	40.0% (12/30)	16.7% (5/30)	/	/	19.0% (4/21)

Overall categorial agreement was 97%. Fourteen VME out of 2029 individual measurements were observed, one for enterococci and ampicillin, three for piperacillin/tazobactam and ten for ciprofloxacin. ME were found in 28 cases: one for *S. aureus* and cefoxitin, six for enterococci and vancomycin and 21 for Gram-negative rods (13 for piperacillin/tazobactam, 8 for ciprofloxacin and none for meropenem). Twelve MinE occurred with Gram-negative rods in all tested drugs. Comparison of piperacillin/tazobactam and ciprofloxacin results showed 21 errors each, whereas comparison of meropenem results showed only four errors (no VME) (Table 3). VME rates for the combinations *S. aureus*/cefoxitin, *Enterococcus* spp./vancomycin, *E. coli*/all drugs tested, *K. pneumoniae*/meropenem and *P. aeruginosa*/meropenem were 0%. The VME rates for piperacillin/tazobactam and ciprofloxacin for *K. pneumoniae* and *P. aeruginosa* were above the 3% cutoff. ME rates fell between 0 and 5.9%. For further details, see Table 2. Substance-based error rates were calculated, too, and can be found in Supplementary Table 1.

**Table 3:** Overall and species-related minimum, maximum and median time required until time to RAST (TTR) and time to Vitek (TTV) could be reported for a single positive blood culture bottle in an evaluation of EUCAST developed rapid antimicrobial susceptibility testing (RAST) directly feasible from positive blood culture fluid on total lab automation (TLA, BD Kiestra™) at the Department for Infectious Diseases at the University Hospital Heidelberg, Germany. The manual comparison of TTR and TTV was restricted to 100 blood culture bottles (20 per species). For the individual positive blood culture bottle, a median TTR of 19:42 h, a median TTV of 37:47 h and a reduced time to report of 17:30 h could be achieved. (*TTR*, time to RAST; *TTV*, time to Vitek)

TTR	TTV	Reduced time to report	TTR	TTV	Reduced time to report	TTR	TTV	Reduced time to report	TTR	TTV	Reduced time to report	TTR	TTV	Reduced time to report	TTR	TTV	Reduced time to report
6:57	24:39	14:13	9:51	25:17	14:13	8:22	24:39	14:47	6:57	25:57	16:30	14:31	32:01	16:43	15:00	32:00	16:15
52:57	71:42	42:30	42:07	60:37	23:00	31:36	55:06	40:30	35:33	69:33	42:30	52:57	71:42	24:43	44:30	60:45	21:43
19:42	37:47	17:30	16:56	34:47	17:43	17:28	35:14	17:08	18:19	36:15	17:36	27:00	44:30	17:30	20:09	39:09	17:37

### Times to report

We analysed the performance data of a total of 100 bottles with 20 isolates per species. Overall, the shortest time to RAST was 6:57 h compared with a maximum of 52:57 h and a median time to RAST of 19:42 h (Table 3). The minimum time to a Vitek report was 24:39 h compared with a maximum of 71:42 h and a median time to Vitek of 37:47 h. The median reduction in time to report in an individual bottle was 17:30 h (minimum: 14:13 h, maximum: 42:30 h).

## Discussion

According to a recent study, less than 5% of European laboratories transmit bacterial identification and AST of positive blood culture to the clinicians 24 h per day and 21.6% of laboratories generally do not forward these findings on Sundays [16]. These results emphasise the urgent need for same-day-results. Of note, a rapid AST is available ≤ 8 h, whereas an ultra-rapid AST is available in ≤ 4 h [16–18]. The aim of this study was to evaluate EUCAST RAST breakpoints for positive blood cultures and to determine the impact of RAST on time to report in a routine laboratory routine.

The composition of the study specimens was comparable with previously published studies, i.e. 10.4% blood culture bottles signalled positive [19]. The time to positivity of 74.0%, 91.6% and 96.7% within 24 h, 48 h and 72 h differed from positivity rates found by other studies. In fact, it is assumed that only a minority positive blood culture is positive within the first 24 h which is actually with febrile neutropenia [20]. However, it has also been proven that many pre-analytical factors such as broth media and atmosphere, blood volume, transportation time, commenced antimicrobial therapy and peripheral or central blood sample may have a great influence on TTP [21].

Introducing RAST using total lab automation reduced time to report of bacterial identification and AST. For our laboratory, choosing 6-h growth was rational regarding rapid and same-day RAST results for frequently detected bacteria in blood cultures that could have not been possible with 8-h growth. Clinical breakpoints for 4-h growth are not available for all tested pathogen, which is why we refused to introduce this time into our laboratory. Of note, all timepoints and time intervals were calculated from the timepoint of positivity. Hence, delay in subculturing and preparing Gram slides, e.g. due to positive signal outside our service period, were included. An optimal automatic processing of an individual positive blood culture allowed same-day-results (minimum TTR: 6:57 h), while Vitek required at least 24:39 h. With it, RAST with 6-h growth fulfilled the criterion for rapid AST [17]. The median reduced time to report of 17:30 h is a prerequisite for an early evidence-based antibiotic treatment, decreased mortality and shortened length of hospital stay for patients [19, 22]. Prolonged TTR and TTV (Table 3) were often attributable to positive blood culture bottles sent from external hospitals with on-site incubators and reaching our laboratory earliest in the forenoon. Hence, images were taken after service hours and read the next day. This lead to delayed reading of RAST and to delayed preparation of ID and Vitek and extended TTRs and especially extended TTVs. In some cases, technical issues, e.g. abort of Vitek2 panels, but also weekends and public holidays with a shortened service period, lead to prolonged times to report. However, due to processing positive bottles with a TLA a 6-h image was available for each and every bottle and enabled reading of RAST a day earlier than MIC results. Therefore, we concluded that for our laboratory, the attachment of external hospital is not an exclusion criterion for applying RAST. Rather the opposite, even external hospitals could benefit from this rapid procedure. However, we believe that aforementioned TTR results were only possible with the automation and it remains interesting, if similar time frames can be achieved in non-automated laboratories in Europe. Still, a fair comparison between TTR and TTV can only be made in a 24/7 working laboratory, whereby we strongly believe that RAST will report faster than MIC results. TLA system includes a closed incubation system which might have positively impacted the cultural growth. In fact, in a study, we were able to prove that the requisite 24-h incubation time for microbial pathogens to reach sufficient growth for susceptibility testing and identification could be shortened by the implementation of TLA compared with the use of conventional methods [23, 24]. Since we could find out that RAST has huge benefits in patient care and efficient therapy, we plan to expand our operational timings in order to cover more blood cultures and to be capable of reporting more RAST, particularly of blood culture signalling positivity during mid-day. In doing so, we also intend to cover more positive blood cultures during our service period and to improve our TTR.

Looking at the categorial agreement, RAST proved reliable. According to the Cumitech definition of VME, ME and MinE, RAST proved reliable for many of the tested antibiotics. For staphylococci/cefoxitin and Gram-negative rods/meropenem, excellent accordance between RAST and MIC results was observed. Regarding enterococci, a ME rate of 4.2% exceeded the suggested 3% and we initially overestimated our VRE cases. Only a single ME case was found with staphylococci. Since its associated blood culture bottle was reported correctly, this error may be due to incorrect execution of subculturing, like a too high inoculum or poor application of antibiotic disc on MHE agar. The same applied to the single VME case of *Enterococcus* spp. for ampicillin.

Data for RAST and meropenem were excellent for all three Gram-negative species. However, results for piperacillin/tazobactam and ciprofloxacin were characterised by high ATU and VME rates. At least for piperacillin/tazobactam, a suboptimal performance with rapid methods was already reported [25]. Since we compared direct-from-specimen agar diffusion with a colony-based MIC determination reference method, this might have added to the error rate. However, broth microdilution for each positive blood culture is not feasible in our daily routine due to the daily received large amount of positive blood culture. Hence, it is also recommended to compare RAST with broth dilution. The same disturbance in our lab routine was considered for Vitek, Phoenix and Micro Scan for direct AST. Furthermore, these systems proved poor in identifying Gram-positive bacteria, which was, as shown in our study, one of our most common found pathogen in bloodstream infections [11]. Additionally, if mixture was not detected in the Gram stain, Vitek was done unnecessarily. Machen et al. also demonstrated that median time from positive bottle to ID and AST was 9.9 h using the combined lysis-filtration method with Vitek MS and which was way longer than RAST [26].

Flexibility in the choice of antibiotics for RAST is a great advantage compared with preformed commercially available AST panels. Furthermore, RAST is compatible with WHO ASSURED criteria (affordable, sensitive, specific, user-friendly, rapid and robust, equipment free, deliverable to users) [18].

The main limitation of our study is the low abundance of highly resistant organisms (MRSA, VRE, CRE) in Germany compared with other countries. Therefore, the extrapolation of our data to high prevalence areas must be done cautiously. Furthermore, with Vitek2 as a reference method, we used a method which is based on a different testing method. Broth microdilution might have been a better reference standard but due to the high number of positive blood cultures an additional testing with microdilution was not feasible in our laboratory. Hence, the unequal comparison between RAST and Vitek2 has to be considered as another limitation.

Additionally, we did not collect clinical information on the patient’s outcome and the impact of RAST on the physician’s therapeutic decision. Pilmis et al. demonstrated that due to fast AST 44% of antibiotic treatment could be modified [27]. However, they used a non-EUCAST compliant RAST-procedure. Analysis of the clinical benefit of RAST would be of huge interest and we plan to do such a study in the near future. Another limitation was found in the limited data comparing closed incubators with digital plate reading to traditional incubation and manual reading.

## Conclusion

Categorial agreement between RAST and Vitek2 exceeded 97%. Inhibition zone interpretation from RAST perfectly correlated to MIC results for cefoxitin and meropenem and with limits for vancomycin, i.e. in antibiotics defining highly resistant bacteria, and hence, reliable results for multi-resistance could be reported. Piperacillin/tazobactam and ciprofloxacin did not fulfil the < 3% VME rates as recommended by Cumitech. We highly recommend the development of RAST breakpoints for further species, particularly for frequently occurring *Enterobacterales* and coagulase-negative staphylococci.

In conclusion, we succeeded in our aim to report AST rapidly and reliably, particularly for MRSA, VRE and CRE. Simultaneously, we were capable of reducing median time to report by 17:30 h even to our external hospitals due to the automatic imaging procedure of TLA. Therefore, RAST combined with TLA could not only contribute to an efficient patient benefit but also accelerated our blood culture procedure.

## Electronic supplementary material

ESM 1 (DOCX 24 kb).
