# Supplementary material for: Evaluation of EUCAST rapid antimicrobial susceptibility testing (RAST) for positive blood cultures in clinical practice using a total lab automation
Source: Eur J Clin Microbiol Infect Dis. 2020 Feb 28;39(7):1305–13. doi: 10.1007/s10096-020-03846-3 (PMC7303068; doi:10.1007/s10096-020-03846-3)
Supplement: Supplementary file 1 — (DOCX 24 kb). [file 10096_2020_3846_MOESM1_ESM.docx]

|  | RAST interpretation | | | Vitek MIC results | | | Errors | | |
| --- | --- | --- | --- | --- | --- | --- | --- | --- | --- |
|  | **S** | **ATU** | **R** | **S** | **I** | **R** | **VME** | **ME** | **MinE** |
| Cefoxitin  (n=221) | **95.0%** (210/221) | **≤1%**  (1/221) | **4.5%**  (10/221) | **95.9%** (212/221) | **/** | **4.1%** (9/221) | **/** | **≤1%**  (1/212) | **/** |
| Ampicillin (n=211) | **34.6%** (73/211) | **/** | **65.4%** (138/211) | **34.1%** (72/211) | **/** | **65.9%** (139/211) | **≤1%**  (1/139) | / | **/** |
| Vancomycin  (n=211) | **64.9%**  (137/211) | **/** | **35.1%** (74/211) | **67.8%** (143/211) | / | **32.2%** (68/211) | **/** | **4.2%** (6/143) | **/** |
| Piperacillin/ Tazobactam  (n=462) | **46.3%** (214/462) | **40.5%** (187/462) | **13.2%** (61/462) | **84.4%** (390/462**)** | **2.2%** (10/463) | **13.4%** (62/462) | **4.8%**  (3/62) | **3.3%** (13/390) | **1.8%** (5/275) |
| Ciprofloxacin (n=462) | **67.1%** (310/462) | **10.4%** (48/462) | **22.5%** (104/462) | **74.2%** (343/462) | **1.2%** (5/462) | **24.6%** (114/462) | **8.8%** (10/114) | **2.3%** (8/343) | **≤1%** (3/414) |
| Meropenem (n=462) | **96.3%** (445/462) | **2.0%** (9/462) | **1.7%** (8/462) | **96.3%** (445/462) | **2.6%** (12/462) | **1.1%** (5/462) | **/** | **/** | **≤1%** (4/453) |

## Supplement

**Supplementary Table 1 Interpretation of EUCAST developed rapid antimicrobial susceptibility testing (RAST) directly feasible from positive blood culture for each tested antibiotic at the Department for Infectious Diseases at the University Hospital Heidelberg, Germany.** A total of 894 positive blood culture bottles were analysed of which 2029 antibiotic measurements were investigated. Very major errors (VME), major errors (ME) and minor errors (MinE) were determined by comparing inhibition zones from RAST to MIC results obtained from Vitek2. Since the EUCAST inhibition zone interpretation guidelines for RAST do not contain an intermediate response, MinE could only arise by comparing intermediate response in Vitek2 to susceptible and resistant response in RAST. Zone diameter that could not be interpreted as susceptible or resistant, were categorised as ‘area of technical uncertainty’ (ATU). ATU categorised isolates were excluded from VME, ME and MinE analysis. (S=susceptible; R=resistant; ATU=area of technical uncertainty)
